# Circulating High-Molecular-Weight (HMW) Adiponectin Level Is Related with Breast Cancer Risk Better than Total Adiponectin: A Case-Control Study

**DOI:** 10.1371/journal.pone.0129246

**Published:** 2015-06-12

**Authors:** Ming-ming Guo, Xue-ning Duan, Shu-de Cui, Fu-guo Tian, Xu-chen Cao, Cui-zhi Geng, Zhi-min Fan, Xiang Wang, Shu Wang, Hong-chuan Jiang, Jian-guo Zhang, Feng Jin, Jin-hai Tang, Hong Liang, Zhen-lin Yang, Hai-bo Wang, Qi-tang Wang, Guo-lou Li, Liang Li, Shi-guang Zhu, Wen-shu Zuo, Li-yuan Liu, Lu Wang, Dan-dan Ma, Shu-chen Liu, Yu-juan Xiang, Lu Liu, Chun-miao Ye, Wen-zhong Zhou, Fei Wang, Li-xiang Yu, Zhong-bing Ma, Zhi-gang Yu

**Affiliations:** 1 School of Medicine, Shandong University, Jinan, Shandong, China; 2 Department of Breast Surgery, the Second Hospital of Shandong University, Jinan, Shandong, China; 3 Breast Disease Center, Peking University First Hospital, Beijing, China; 4 Department of Breast Surgery, Affiliated Tumor Hospital of Zhengzhou University, Zhengzhou, Henan, China; 5 Department of Breast Surgery, Shanxi Cancer Hospital, Taiyuan, Shanxi, China; 6 National Clinical Research Center for Cancer, Tianjin Medical University Cancer Institute and Hospital, Tianjin, China; 7 Breast Center, the Fourth Hospital of Hebei Medical University, Shijiazhuang, Hebei, China; 8 Department of Breast Surgery, the First Hospital of Jilin University, Changchun, Jilin, China; 9 Department of Breast Surgery, Cancer Hospital, Chinese Academy of Medical Sciences, Beijing, China; 10 Breast Disease Center, Peking University People's Hospital, Beijing, China; 11 Department of General Surgery, Beijing Chaoyang Hospital, Beijing, China; 12 Department of General Surgery, the Second Affiliated Hospital of Harbin Medical University, Harbin, Heilongjiang, China; 13 Department of Breast Surgery, the First Affiliated Hospital of China Medical University, Shenyang, Liaoning, China; 14 Department of General Surgery, Nanjing Medical University Affiliated Cancer Hospital Cancer Institute of Jiangsu Province, Nanjing, Jiangsu, China; 15 Department of General Surgery, Linyi People’s Hospital, Linyi, Shandong, China; 16 Department of Thyroid and Breast Surgery, the First Affiliated hospital of Binzhou Medical University, Binzhou, Shandong, China; 17 Breast Center, Qingdao University Affiliated Hospital, Qingdao, Shandong, China; 18 Department of Breast Surgery, the Second Affiliated Hospital of Qingdao Medical College, Qingdao Central Hospital, Qingdao, Shandong, China; 19 Department of Breast and Thyroid Surgery, Weifang Traditional Chinese Hospital, Weifang, Shandong, China; 20 Department of Breast and Thyroid Surgery, Zibo Central Hospital, Zibo, Shandong, China; 21 Department of Breast Surgery, Yantai Yuhuangding Hospital, Yantai, Shandong, China; 22 Breast Cancer Center, Shandong Cancer Hospital, Jinan, Shandong, China; 23 Epidemiology Institute, School of Public Health, Shandong University, Jinan, Shandong, China; 24 Division of Epidemiology and Biostatistics, School of Public Health, Shandong University, Jinan, Shandong, China; University of South Alabama Mitchell Cancer Institute, UNITED STATES

## Abstract

The level of total adiponectin, a mixture of different adiponectin forms, has been reported associated with breast cancer risk with inconsistent results. Whether the different forms play different roles in breast cancer risk prediction is unclear. To examine this, we measured total and high molecular weight (HMW) adiponectin in a case-control study (1167 sets). Higher circulating HMW adiponectin was negatively associated with breast cancer risk after adjusting for menopausal status and family history of breast cancer (*P*=0.024). We analyzed the relationship between adiponectin and breast cancer risk in 6 subgroups. Higher circulating HMW adiponectin was also negatively associated with breast cancer risk (*P*=0.020, 0.014, 0.035) in the subgroups of postmenopausal women, negative family history of breast cancer, BMI>=24.0. Total adiponectin was positively associated with breast cancer (*P*=0.028) in the subgroup of BMI<=24.0. Higher HMW/total adiponectin ratio was negatively associated with breast cancer (*P*=0.019) in the subgroup of postmenopausal women. Interestingly, in the subgroup of women with family history of breast cancer, higher circulating total and HMW adiponectin were positively associated with breast cancer risk (*P*=0.034, 0.0116). This study showed different forms of circulating adiponectin levels might play different roles in breast cancer risk. A higher circulating HMW adiponectin is associated with a decreased breast cancer risk, especially in postmenopausal, without family history of breast cancer or BMI>=24.0 subgroups, whereas higher circulating HMW adiponectin levels is a risk factor in women with a family history of breast cancer. Further investigation of different forms of adiponectin on breast cancer risk is needed.

## Introduction

Breast cancer is the most common cancer and the leading causes of cancer death among women worldwide. The latest World Health Organization (WHO) statistics show that there were 1.67 million new breast cancer cases diagnosed in 2012, which comprised 25% of all cancers diagnosed that year[1]. Annual diagnoses of breast cancer in China are now about half of those in the European Union (332 000 in 2008; population 498 million), and are similar to the number of cases in the USA (182 000 cases in 2008; population 304 million)[2]. Many factors contribute the risk of breast cancer including family history, genetics and life-style. One recent factor that has been associated with increased cancer risk is obesity[3]. In fact, we recently reported that a higher body mass index (BMI) was related with high breast cancer risk in Han Chinese women[4].

Adiponectin, an obesity-related protein, has been reported to be associated with obesity, metabolic syndrome, and several types of cancer, including breast cancer. Adiponectin is an adipokine secreted exclusively by adipocytes and exhibits insulin-sensitizing, inflammatory-related, antiatherogenic, proapoptotic, and antiproliferative properties[5–7]. Three configurations of adiponectin have been identified and have been postulated to have distinct biological roles: low molecular weight (LMW), middle molecular weight (MMW) or high molecular weight (HMW) [8,9]. HMW adiponectin is the active form, which may specifically activate the AMPKinase signaling cascade[10]. HMW and MMW adiponectin activate NF-κB, but LMW adiponectin does not [11]. In contrast, the affinity between the adiponectin species and the adiponectin receptors (AdipoRs) is not the same. Of the three AdipoRs, AdipoR1 is the high-affinity receptor for globular adiponectin which is a smaller fragment generated by proteolytic cleavage of full-length adiponectin; AdipoR2 has intermediate affinity for all adiponectin forms [12,13]. T-cadherin, which is associated with downstream effector molecules and may serve as a coreceptor for adiponectin, is the receptor for HMW adiponectin, but not for the LMW form[14]. Together this suggests that the HMW may be the important form in pathogenesis.

Interestingly, results from previous epidemiologic studies for the association between adiponectin and breast cancer risk have been inconsistent, with the reason unknown. For example, in three meta-analyses regarding the relationship between adiponectin levels and breast cancer risk published in the last two years, one suggested that low circulating adiponectin levels were associated with an increased breast cancer risk[15], whereas the other two (including our own meta-analysis) showed that high adiponectin levels might decrease breast cancer risk, specifically in postmenopausal women[16,17]. It is worth mentioning that the reagents used in the studies were recombinant human or mouse adiponectin, which are always purified single compound, but in the human body adiponectin can homotrimerize and the trimers can polymerize into large complexes. Among the different forms, HMW adiponectin may mediate the majority of adiponectin’s effects in endothelial cells[18].

Here, we hypothesized that HMW adiponectin or the HMW/total adiponectin ratio, but not total adiponectin, associated with risk of breast cancer, which may partly explain the inconsistent results from literature. To test our hypothesis, we did a large 1:1 case-control study of 1167 cases from 21 hospitals from northern China. We investigated plasma total adiponectin, HMW adiponectin and the HMW/total adiponectin ratio, and their association with breast cancer risk in the context of other known risk factors.

## Materials and Methods

### Study subjects

We recruited female patients from 21 hospitals in 11 provinces in northern and eastern China from April 2012 to April 2013. The inclusion criteria for breast cancer cases were: (1) newly diagnosed and histologically confirmed breast cancer; (2) Han ethnic group; (3) age 25 to 70 years old. For the control group, the following criteria were used: (1) physical examination results were negative; (2) ultrasound scans of breast and/or mammographic screening results were negative; (3) matched age with the cases (±3 years); (4) women who had been hospitalized or had a regular physical examination in the same hospital with matched case in the same time period (±2 months); (5) no evidence of cancer or history of cancer; (6) Han ethnic group. Anyone who had a neoplastic disease at any other site, history of cancer, or other major chronic disease, was excluded from the study. Cases and controls were matched based on age, hospital, and timing of examination at 1:1 ratio. There were 1167 case-control sets included in this study.

### Ethics Statement

The study protocol and procedures were approved by the Institutional Review Boards at the Second Hospital of Shandong University. All participants have provided their written informed consent.

### Data collection

Data on breast cancer risk factors was obtained by in-person interviews based on a self-designed structured questionnaire. The questionnaire contained six aspects: (1) demographic characteristics and female physiological and reproductive factors, such as age, height, weight, age at menarche, age at menopause; (2) medical and family history: primarily, breast-related diseases and family history of breast cancer; (3) lifestyle habits: smoking (including passive smoking), alcohol intake, dietary habits; (4) medication and chemical exposure history: hair dyes, antidiabetic agents; (5) breast cancer-related knowledge: risk factors for breast cancer, early signs and symptoms of breast cancer; (6) medical record: specifically, information gathered from the clinical breast examination, which included the results from visual examination, palpation and related diagnostic tests. The histological and immunohistochemical diagnosis of breast cancer patients were also collected from the medical record

Peripheral blood samples were obtained after fasting at least 8 hours early in the morning. Blood samples of the cases were obtained pre-operation and pre-therapeutic intervention, including neoadjuvant chemotherapies, radiation ad hormone therapies.

**Fig 1:**
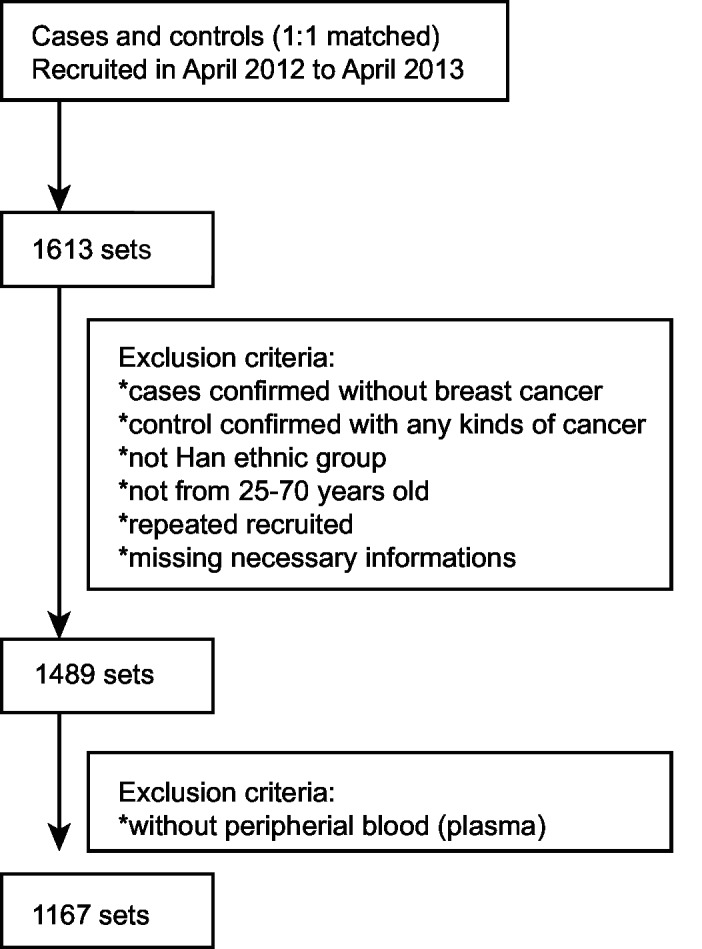
Flow Diagram of the Study Subjects Selection.

After excluding subjects with inadequate information or samples, 1167 sets of cases and controls met our inclusion criteria (Fig 1).

### Laboratory analyses

Total and HMW adiponectin levels were assayed from plasma using a human total adiponectin and HMW adiponectin quantitative ELISA kits with sensitivity of 0.079–0.891ng/ml, 0.086–0.989ng/ml, respectively (RD systems, SRP300, SHWAD0). Each sample was assayed twice and the average of the data was used. No samples were below the detection limits. All analyses were performed at the Central Research Laboratory, the Second Hospital of Shandong University (Jinan, China) according to the manufactory recommended protocols. Data were collected in an case-control status blind manor.

### Statistical analysis

All statistics except the ROC curve were performed by SAS Statistical Software (Version 9.1.3; SAS Institute, Cary, NC, USA), and the ROC estimate were calculated through MedCalc. Descriptive characteristics of the group variables are expressed as mean (±standard deviation, SD). The *P*-values of continuous variables were determined by Student’s *t*-tests and the *P*-values of categorical variables were determined by Chi-Square tests. HMW adiponectin level, HMW/total adiponectin ratio were divided into two class based on a cut-off value as described below. The odds ratio (ORs) and 95% confidence intervals (CIs) were obtained by using single and multiple binary logistic regression analyses. The models were adjusted for BMI, age, diabetes mellitus, age at menarche, etc. All *P*-values were two-sided and *P*<0.05 was considered significant.

The cut-offs for the total adiponectin, HMW adiponectin and HMW/total adiponectin ratio were assessed for the overall and corresponding subgroup by receiver operating characteristic (ROC) curve analysis, in which the sensitivity (SE) is plotted as a function of 1-specificity (1-SP). The Youden Index (*J*), one of the main summary statistics of the ROC curve, defines the maximum potential effectiveness of a biomarker. J can be formally defined as *J* = max_*c*_{SE (*c*)+SP (*c*)-1}. The cut-off value that achieves this maximum is referred to as the optimal cut-off point that optimizes the biomarker’s discriminating power when the sensitivity and specificity bear equal weight[19].

## Results

The mean age (±SD) of cases is 47.65 (±8.65); the mean age (±SD) of controls is 46.72 (±8.77). The characteristics of cases and controls were shown in Table 1. The BMI is higher in cases compared with controls. The number of postmenopausal women or those with a family history of breast cancer is more in cases compared with controls. No differences between case and control groups were observed in the following six factors: number of births, age at first birth, number of children, height, age at menarche and diabetes mellitus.

**Table 1:** Basic Characteristics of Cases and Controls.

	Case	Control	*P*
	Mean ± SD or n (%)	Mean ± SD or n (%)	
Number of births	1.97± 5.25	1.75± 5.26	0.308 ^a^
Age at first birth(years)	24.71± 6.03	24.90± 6.46	0.485 ^a^
Number of children	1.89± 4.52	1.72± 5.18	0.402 ^a^
Height(cm)	160.00± 4.81	160.30± 4.33	0.269 ^a^
BMI (kg/m2)			0.003^b^
<24.0	541 (48.43)	577(51.20)	
24.0–28.0	428 (38.32)	452 (40.11)	
>28.0	148 (13.25)	98 (8.70)	
n/a	50	40	
Age at menarche(years)			0.648^b^
≤12	86(7.53)	92(8.04)	
>12	1056(92.47)	1052(91.96)	
n/a	25	23	
Menopause status			0.021^b^
Yes	396 (34.71)	339 (30.16)	
No	745 (65.29)	785 (69.84)	
n/a	26	43	
Diabetes Mellitus			0.543^b^
Yes	53 (4.60)	47 (4.08)	
No	1100 (95.40)	1105 (95.92)	
n/a	14	15	
Family history of BC			0.004^b^
Yes	76 (6.51)	45 (3.86)	
No	1091 (93.49)	1122 (96.14)	

SD, standard deviation; BC, breast cancer; BMI, body mass index; n/a, not available;

^a^, t test;

^b^, chi-square test.

We evaluated the status of circulating adiponectin levels and the different subgroups as a continuous number factors (Table 2) to further investigate the role of adiponectin in breast cancer risk. Circulating total adiponectin, HMW adiponectin and the HMW/total adiponectin ratio were not significant different between case and controls. However, the circulating HMW/total adiponectin ratio levels were found to be significantly higher in the controls compared to cases among the postmenopausal women (*P* = 0.016). In addition, the circulating total and HMW adiponectin levels were significantly lower in controls compared to cases among those women with a family history of breast cancer (*P* = 0.049, 0.032).

**Table 2:** The Status of Plasma Adiponectin Level in 1167 Sets and Subgroup.

Factors	Case	Control	*P*
	mean ± SD	mean ± SD	
All patients	n = 1167	n = 1167	
Total adiponectin (μg/ml)	6.34(3.54)	6.56(3.72)	0.150
HMW adiponectin (μg/ml)	2.52(1.88)	2.58(1.88)	0.403
HMW/total adiponectin ratio	0.38(0.15)	0.38(0.15)	0.590
Postmenopausal women	n = 396	n = 339	
Total adiponectin (μg/ml)	6.58 (3.74)	6.95 (4.08)	0.201
HMW adiponectin (μg/ml)	2.59 (2.01)	2.84 (2.14)	0.113
HMW/total adiponectin ratio	0.37 (0.15)	0.40 (0.15)	0.016
Non-postmenopausal women	n = 745	n = 785	
Total adiponectin (μg/ml)	6.24 (3.46)	6.44 (3.57)	0.232
HMW adiponectin (μg/ml)	2.49 (1.83)	2.49 (1.76)	0.410
HMW/total adiponectin ratio	0.38 (0.16)	0.38 (0.15)	0.660
Positive family history of BC	n = 76	n = 45	
Total adiponectin (μg/ml)	6.37 (3.42)	5.18 (2.74)	0.049
HMW adiponectin (μg/ml)	2.92 (1.95)	2.18 (1.54)	0.032
HMW/total adiponectin ratio	0.44 (0.13)	0.42 (0.16)	0.480
Negative family history of BC	n = 1091	n = 1122	
Total adiponectin (μg/ml)	6.34 (3.55)	6.62 (3.74)	0.081
HMW aidponectin (μg/ml)	2.49 (1.87)	2.60 (1.89)	0.832
HMW/total adiponectin ratio	0.38 (0.16)	0.38 (0.15)	0.714
BMI<24.0	n = 541	n = 577	
Total adiponectin (μg/ml)	6.85 (3.60)	6.99 (3.87)	0.091
HMW aidponectin (μg/ml)	2.81 (1.95)	2.81 (2.03)	0.992
HMW/total adiponectin ratio	0.39 (0.16)	0.39 (0.16)	0.974
BMI> = 24.0	n = 576	n = 550	
Total adiponectin (μg/ml)	5.88 (3.39)	6.13 (3.56)	0.261
HMW aidponectin (μg/ml)	2.23 (1.77)	2.34 (1.68)	0.738
HMW/total adiponectin ratio	0.36 (0.15)	0.37 (0.15)	0.951

BC, breast cancer; BMI, body mass index; SD, standard deviation.

Based on the above results, we chose to evaluate the effectiveness of total adiponectin, HMW adiponectin and HMW/total adiponectin ratio by performing ROC curve analysis to get best cutoff value. In the overall data, the area under curve (AUC) was 0.515 and the cutoff value of 1.42 μg/ml of HMW adiponectin (*J*: 0.039) significantly discriminated the cases from controls (*P* = 0.024, OR = 0.815, 95%CI, 0.682–0.973) after adjusting for menopause and family history of breast cancer (Table 3). In the postmenopausal subgroup, the AUC was 0.540 and the cutoff value of 1.98 μg/ml of HMW adiponectin (*J*: 0.091) significantly discriminated the cases from controls (*P* = 0.020, OR = 0.706, 95%CI, 0.527–0.947) (Table 4). In the subgroup of negative family history of breast cancer, the AUC was 0.523 and the cutoff value of 1.42 μg/ml of HMW adiponectin (*J*: 0.048) significantly discriminated the cases from controls after adjusting for menopausal status (*P* = 0.007, OR = 0.779, 95%CI, 0.650–0.935) (Table 5). In the subgroup of positive family history of breast, the AUC was 0.625 and the cutoff value of 3.20 μg/ml of HMW adiponectin (*J*: 0.201) significantly discriminated the cases from controls (*P* = 0.011, OR = 3.350, 95%CI, 1.322–8.486) (Table 5). In the subgroup of BMI> = 24.0, the cutoff value of 1.42 μg/ml of HMW adiponectin also significantly discriminated the cases from controls (*P* = 0.035, OR = 0.771, 95%CI, 0.605–0.982) (Table 6). Only in the subgroup of postmenopausal women, HMW/total adiponectin ratio, the AUC was 0.553 and the cut-off value of 0.38 HMW/total adiponectin (*J*: 0.095) significantly discriminated the cases from controls (*P* = 0.019, OR = 0.705, 95%CI, 0.526–0.944) (Table 4).

**Table 3:** Association Between Plasma Adiponectin and Breast Cancer by Logistic Regression.

Factors	OR	95%CI	*P*
Univariate model			
menopause (no = reference)	1.231	1.032–1.468	0.021
BMI (<24.0;24.0–28.0; > = 28.0, <24.0 is reference)	1.174	1.038–1.328	0.010
Diabetes mellitus (no = reference)	1.133	0.758–1.692	0.543
Family history of BC (no = reference)	1.737	1.190–2.535	0.004
age at menarche (< = 12 years old is reference)	0.931	0.686–1.265	0.648
Total adiponectin (< = cut-off value is reference)	1.071	0.902–1.272	0.431
HMW adiponectin (c< = cut-off value is reference)	0.835	0.701–0.993	0.042
HMW/total adiponectin ratio (< = cut-off value is reference)	0.923	0.784–1.087	0.338
Multivariate model^a^			
HMW adiponectin (< = cut-off value is reference)	0.815	0.682–0.973	0.024

^a^, adjusting by menopause status and family history of breast cancer; the cut-off value for total adiponectin, HMW adiponectin and HMW/total adiponectin ratio are 4.46μg/ml, 1.42μg/ml and 0.39.

**Table 4:** Association Between Plasma Adiponectin and Breast Cancer in Postmenopausal and Non-postmenopausal Subgroup by Univariate Logistic Regression.

Factors	Postmenopausal women^a^			Non postmenopausal women^b^		
	OR	95%CI	*P*	OR	95%CI	*P*
BMI (< = 24.0;24.0–28.0; >28.0, < = 24.0 is reference)	1.025	0.834–1.260	0.813	1.303	1.113–1.524	0.001
Diabetes mellitus (no = reference)	1.031	0.624–1.702	0.906	1.211	0.587–2.499	0.605
Family history of BC (no = reference)	1.631	0.838–3.177	0.150	1.772	1.112–2.823	0.016
Age at menarche (< = 12 years old = reference)	0.692	0.365–1.314	0.260	0.908	0.634–1.301	0.600
Total adiponectin (< = cut-off value is reference)	0.683	0.466–1.001	0.051	0.814	0.662–1.000	0.050
HMW adiponectin (< = cut-off value is reference)	0.706	0.527–0.947	0.020	0.865	0.703–1.064	0.170
HMW/total adiponectin ratio (< = cut-off value is reference)	0.705	0.526–0.944	0.019	0.851	0.446–1.621	0.623

BMI, body mass index;

^a^, for postmenopausal women;

^b^, for non-postmenopausal women; BC, breast cancer; the cut-off values are: for total adiponectin, a = 3.30μg/ml, b = 6.59μg/ml; for HMW adiponectin, a = 1.98μg/ml, b = 1.62μg/ml; for HMW/total adiponectin ratio, a = 0.38, b = 0.09.

**Table 5:** Association Between Plasma Adiponectin and Breast Cancer in Family History of Breast Cancer Subgroup by Univariate and Multivariate Logistic Regression.

Factors	Positive family history of BC^a^			Negative family history of BC^b^		
	OR	95%CI	*P*	OR	95%CI	*P*
Univariate model						
Menopause (no = reference)	1.137	0.515–2.512	0.751	1.235	1.030–1.480	0.022
BMI (< = 24.0;24.0–28.0; >28.0, < = 24.0 is reference)	0.749	0.411–1.366	0.346	1.208	1.065–1.370	0.003
Diabetes mellitus (no = reference)	3.088	0.349–27.353	0.311	1.077	0.706–1.642	0.731
Age at menarche (< = 12 years is reference)	0.396	0.128–1.228	0.109	1.020	0.742–1.403	0.903
Total adiponectin (< = cut-off value is reference)	2.265	1.064–4.820	0.034	0.846	0.713–1.003	0.053
HMW adiponectin (< = cut-off value is reference)	3.350	1.322–8.486	0.011	0.799	0.669–0.956	0.014
HMW/total adiponectin ratio (< = cut-off value is reference)	0.827	0.387–1.766	0.623	0.891	0.753–1.054	0.177
Multivariate model^c^						
HMW adiponectin (< = cut-off value is reference) ^b^	—	—	—	0.779	0.650–0.935	0.007

BMI, body mass index;

^a^, for postmenopausal subgroup;

^b^, for non-postmenopausal subgroup;

^c^, adjusting by menopause status; the cut-off values are: for total adiponectin, a = 5.23μg/ml, b = 6.59μg/ml; for HMW adiponectin, a = 3.20μg/ml, b = 1.42μg/ml; for HMW/total adiponectin ratio, a = 0.47,b = 0.39.

**Table 6:** Association Between Plasma Adiponectin and Breast Cancer in BMI Subgroup by Univariate Logistic Regression.

Factors	BMI<24.0^a^			BMI> = 24.0^b^		
	OR	95%CI	*P*	OR	95%CI	*P*
Menopause (no = reference)	1.383	1.065–1.796	0.015	1.049	0.818–1.345	0.704
Diabetes mellitus (no = reference)	1.631	0.778–3.419	0.195	0.944	0.578–1.542	0.819
Family history of BC (no = reference)	2.132	1.270–3.582	0.004	1.354	0.753–2.433	0.311
Age at menarche (< = 12 years old = reference)	1.011	0.643–1.590	0.962	1.180	0.765–1.821	0.455
Total adiponectin (< = cut-off value is reference)	0.629	0.416–0.950	0.028	0.813	0.632–1.045	0.106
HMW adiponectin (< = cut-off value is reference)	0.720	0.463–1.120	0.145	0.771	0.605–0.982	0.035
HMW/total adiponectin ratio (< = cut-off value is reference)	0.992	0.784–1.255	0.946	0.828	0.653–1.052	0.122

BMI, body mass index;

^a^, for BMI<24.0;

^b^, for BMI> = 24.0; the cut-off values are: for total adiponectin, a = 12.52μg/ml, b = 6.82μg/ml; for HMW adiponectin, a = 5.61μg/ml, b = 1.42μg/ml; for HMW/total adiponectin ratio, a = 0.37, b = 0.39.

## Discussion

In the present study we have demonstrated the value of measuring circulating HMW adiponectin in breast cancer. Our results show that higher HMW adiponectin level is negatively associated with breast cancer risk in Chinese women. This association is independent of menopausal status and family history of breast cancer. This is in accordance with the result of a case-control investigating pheochromocytoma[20]. Higher HMW adiponectin level is also negatively associated with breast cancer in postmenopausal, negative family history of breast cancer or BMI> = 24.0 subgroup. But, both total and higher HMW adiponectin levels are positively associated with breast cancer in positive family history of breast cancer. G84R, G90S, G25D, G28E mutations of *ADIPOQ* could affect the formation of HMW adiponectin, which resulted in impairment of its complement activation which means that some mutation of *ADIPOQ* might affect the circulating level of HMW adiponectin and its biological function[21]. Certain mutation of *ADIPOQ* may be the true relationship between HMW adiponectin and breast cancer risk for women who have positive family history of breast cancer in China. The number of women who have family history of breast cancer in this study is only 121/2334 (cases n = 76, controls n = 45), which also limits the power of our analysis. Further studies are needed to confirm the relationship between HMW adiponectin and breast cancer risk especially in women who have a family history of breast cancer in China.

The mechanisms of how HMW adiponectin impact the risk of breast cancer is not clear. With respect to inflammatory-related responses, HMW adiponectin is responsible for proinflammatory actions, whereas LMW adiponectin is responsible for anti-inflammatory actions[22]. More importantly, for cancers, HMW adiponectin were three times higher in patients with pheochromocytoma than in controls, yet lower HMW adiponectin was an independent risk factor for worse hepatocellular carcinoma histological grades and HMW adiponectin was decreased in patients with metastatic renal cancer compared with those with localized one. The role of HMW adiponectin in breast cancer is also being actively investigated [20,23,24]. For example, in a hospital-based case-control study of 74 female breast cancer patients and 76 controls, HMW was shown to be strongly and inversely associated with breast cancer risk, independent of classical risk factors such as leptin and the IGF-1 system[25]. Importantly, this study did not offer any insight into the HMW/total adiponectin ratio levels.

Similarly, we also show the value of the HMW/total adiponectin ratio in breast cancer risk. We found that a higher HMW/total adiponectin ratio is a protective factor of breast cancer in postmenopausal women. One possible explanation for the observed changes in the HMW/total adiponectin ratio could be due to polymorphisms in Cys36 of *ADIPOQ*, which is considered to be the key amino acid for the formation of HMW adiponectin[8,22]. The HMW/total adiponectin ratio reflects the component of the circulating adiponectin complex. Previous reports have highlighted the importance of a patient’s HMW/total adiponectin ratio, rather than their total adiponectin level. For example, changes in the HMW/total adiponectin ratio are closely associated with improvements in insulin sensitivity during thiazolidinedione treatment in humans, but not for changes in total adiponectin’s concentration [26]. In addition, HMW/total adiponectin ratio has been reported to be a better predictor of type 2 diabetes risk compared with total adiponectin[27,28]. Despite HMW adiponectin being recognized as a predictor of risk for certain diseases and even a stronger predictor than total adiponectin in specific populations, the association between the HMW/total adiponectin ratio and the risk of breast cancer has not been thoroughly investigated.

The results possibly explain the inconsistent epidemiologic results that HMW adiponectin or HMW/total adiponectin ratio may be the real factor related to breast cancer risk, not the total adiponectin complex, and implicate differing pathways toward carcinogenesis, especially in special subgroups of individuals.

## Conclusions

In conclusion, we found that different forms of circulating adiponectin levels may play different roles in breast cancer risk. Higher circulating HMW adiponectin is associated with a decreased breast cancer risk in Chinese women, especially in postmenopausal, without family history of breast cancer or BMI> = 24.0 subgroup. Whereas higher circulating HMW adiponectin levels is a risk factor in women with a family history of breast cancer. Higher HMW/total adiponectin ratio is a protective factor of breast cancer in postmenopausal women. Our data provides additional evidence for a biological link between menopausal status, hereditary factor, BMI and breast cancer, suggesting an independent role of different circulating adiponectin in the carcinogenesis of breast cancer. In addition, because circulating adiponectin can be influenced by a number of behavioral and drug interventions, such as fruit, weight loss, the observation in this study may help guide a preventive strategy for breast cancer[29–31]. Further investigation into the association between HMW adiponectin, HMW/total adiponectin ratio, the participation of the different receptors, and *ADIPOQ* polymorphisms are necessary. In addition, due to differences between risk factors between ethnicities, our findings derived from a Han Chinese population, may not generalize to other ethnic groups.

## Supporting Information

S1 Table Characteristics of Studies about the Relationship Between HMW adiponectin and Breast Cancer Risk. (DOCX)
