# Supplementary material for: Circulating High-Molecular-Weight (HMW) Adiponectin Level Is Related with Breast Cancer Risk Better than Total Adiponectin: A Case-Control Study
Source: PLoS One. 2015 Jun 12;10(6):e0129246. doi: 10.1371/journal.pone.0129246 (PMC4466435; doi:10.1371/journal.pone.0129246)
Supplement: S1 Table — (DOCX) [file pone.0129246.s001.docx]

Supplement Table 1. Characteristics of Studies about the Relationship Between HMW adiponectin and Breast Cancer Risk

| First author | Year | Location | Study design | No. of case/control | *P* | OR | 95%CI | Conclusion |
| --- | --- | --- | --- | --- | --- | --- | --- | --- |
| Minatoya M | 2014 | Japan | Case-control study | 22/22^a^ | 0.001 | 0.01 | 0.00-0.26 | I |
| Minatoya M | 2014 | Japan | Case-control study | 44/44^b^ | 0.014 | 0.13 | 0.03-0.57 | I |
| Minatoya M | 2013 | Japan | Case-control study | 63/76 | 0.001 | 0.09 | 0.03-0.33 | I |
| Antje Körner | 2007 | Greece | Case-control study | 74/76 | 0.05 | 0.30 | 0.11-0.82 | I |

a, for premenopausal women; b, for postmenopausal women; I, higher HMW adiponectin is protective factor for breast cancer.
